# Dating Violence, Emotional Dependence and Mental Health Problems in Ecuadorian College Students

**DOI:** 10.3390/healthcare14070834

**Published:** 2026-03-25

**Authors:** Mayra Castillo-Gonzáles, Benito León-del-Barco, Santiago Mendo-Lázaro, Emilio Terán-Andrade

**Affiliations:** 1 Department of Psychology, Faculty of Health Sciences, Universidad Internacional SEK, Quito 170125, Ecuador; 2 Department of Psychology, Faculty of Teacher Training College, University of Extremadura, 10071 Cáceres, Spain; bleon@unex.es (B.L.-d.-B.); smendo@unex.es (S.M.-L.); 3 School of Law, Universidad de las Americas (UDLA), Quito 170125, Ecuador; emilio.teran@udla.edu.ec

**Keywords:** dating violence, emotional dependence, mental health, gender, students

## Abstract

**Background:** Dating violence and emotional dependence are consistently associated with mental health problems among university students. However, less is known about how these associations operate indirectly and whether they differ by gender, particularly in Latin American contexts. **Objective:** To evaluate a model examining the association between dating violence (X) and mental health problems (Y) among university students, with emotional dependence (M) as an intervening variable and gender (W) as a moderator. **Methods:** A quantitative, cross-sectional study was conducted with 3202 university students. Participants completed the Dating Violence Questionnaire (CUVINO), the Emotional Dependence Questionnaire (CDE), and the Strengths and Difficulties Questionnaire (SDQ-Cas). Conditional process analyses were performed using Hayes’ PROCESS macro (Model 14) with bootstrapping procedures. **Results:** Dating violence and emotional dependence were significantly associated with internalizing mental health problems. Emotional dependence showed a significant indirect association between dating violence and internalizing problems. This indirect association varied by gender, as indicated by a significant index of moderated mediation. **Conclusions:** The findings support a conditional indirect association between dating violence and internalizing mental health problems through emotional dependence, moderated by gender. These results highlight the importance of considering relational and gender-related factors when examining the mental health correlates of dating violence among university students.

## 1. Introduction

Dating violence (DaV) is a serious problem associated with poor mental health outcomes [1]. Due to its serious consequences, it is considered an urgent and high-priority public health issue [2]. In recent years, numerous studies have attempted to examine and understand DaV among adolescents and young adults. Nonetheless, to this day, defining this problem in this context remains a challenge due to the difficulty of distinguishing it from other forms of partner violence and the diversity of terms and theoretical approaches [3]. Below is a more detailed explanation of DaV.

### 1.1. Contextualization and Prevalence of Dating Violence

Violence in intimate relationships has been categorized as domestic, family, and marital violence, although these definitions are not entirely equivalent [4]. The first study on DaV was conducted by Makepeace in 1981 [5], who emphasized the importance of studying this phenomenon due to its high prevalence and particular characteristics that differ from violence in married couples. Later, in 1989, Sugarman and Hotaling defined this type of violence as the use or threat of physical force with the intent to cause harm; however, this definition was deemed insufficient [6].

Currently, one of the most widely accepted definitions for contextualizing DaV is that proposed by Breiding et al., who describe “intimate partner violence” as any form of physical, sexual, stalking, or psychological aggression committed by a current or former intimate partner [7]. In the context of young couples, the term DaV encompasses various forms of violence that may occur in person or through digital media.

DaV refers to violence occurring between adolescent and young adult couples who live separately and are not married [8]. This means that DaV has distinctive characteristics, such as levels of commitment, relationship duration, sexual intimacy, and conflict resolution strategies. Unlike adult couples, relationships involving violence among college students generally do not involve economic dependence, domestic co-responsibility, or co-parenting [3]. Additionally, DaV often tends to be reciprocal, meaning that both partners may act as both victims and perpetrators. According to Maquibar et al. [9], this violence is often subtle, normalized and part of daily interactions, with psychological violence being the most prevalent. This frequently involves controlling social aspects such as meetings, schedules, and clothing choices.

During adolescence and early adulthood, violent behaviors are often observed in romantic relationships and may co-occur with patterns of dysfunction and changes in relational behavior [3,10]. Adolescent romantic relationships may relate to the satisfaction and quality of later relationships in young adulthood [11,12].

The prevalence of DaV shows considerable variability worldwide and across different types of violence [12,13,14,15]. A systematic review and meta-analysis published in 2024 [14] indicated that, on average, adolescents reported prevalence rates of 20% for physical violence, 30% for psychological violence, and 15% for sexual violence. Gender differences have also been observed, such that women tend to report higher levels of victimization, whereas men report higher levels of perpetration. These patterns are consistent with findings from meta-analyses conducted in African [15] and European populations [13], in which women reported higher levels of DaV victimization compared to men.

In Ecuador, a study conducted with university students [16] indicated that 90.4% of male and 88.1% of female students reported experiencing some form of DaV. When both intensity (mild, moderate or severe) and type of violence were considered, gender differences were observed. Men reported higher frequencies of mild psychological, physical, sexual, and instrumental violence. At the moderate level, psychological violence was more frequently reported by men, whereas women reported higher levels of sexual violence. In cases of severe violence, women reported higher frequencies of psychological and instrumental violence. These findings suggest that the type and severity of victimization are differentially distributed across male and female university students.

Additionally, Castillo and Terán [17] identified a bidirectional pattern of violence among Ecuadorian university students, in which both men and women reported being both victims and perpetrators, engaging in aggressive and controlling behaviors through digital media and exhibiting high levels of digital dating violence. This pattern may be facilitated by characteristics inherent to the university context, such as the intensive use of social networking platforms, increased relational autonomy, reduced adult supervision, and campus social dynamics. However, variability across studies has been reported, as other research indicates that female university students are more likely to experience intimate partner violence [18,19], highlighting the importance of considering cultural, contextual, and gender-related factors when examining dating violence in Ecuadorian university populations.

Within the Ecuadorian context, cultural norms and traditional gender roles have been described as relevant factors in discussions of intimate partner violence [18]. In this regard, Cuadrado and Martín Mora [20], in a comparative study of Spanish and Ecuadorian adolescents, observed that Ecuadorian adolescents reported higher levels of hostile sexism and that nationality was associated with higher rates of victimization in a context characterized by conservative gender norms and religiously influenced cultural values. Research conducted with university students indicates that different forms of DaV are interrelated with socio-emotional and family-related factors. In addition, the bidirectional nature of violence and its associations with variables such as family functioning, age, and socioeconomic status reflect the complexity of these dynamics [21], together with individual characteristics such as low self-esteem and emotional dependence, which are associated with the persistence of elevated levels of this phenomenon in the country [17,18,22].

DaV may be linked to emotional dependence (ED), especially in adolescents and young adults, which influences the dynamics and persistence of these behaviors. Several studies [3,16,17,23,24,25] have found that DaV is closely related to ED, which can significantly aggravate the mental health of young people.

### 1.2. Dating Violence, Emotional Dependence, Mental Health and Gender

ED has been studied in relation to other pathologies such as love addiction and dependent personality disorder. According to Izquierdo and Gómez [26], since ancient times, terms such as “harmful love” or “possessive love” have been used to describe individuals who display intense feelings toward their partner, treating them as an object of possession. Nonetheless, in recent years, the term most commonly used is ED or affective dependency. ED has played an important role in understanding DaV, which explains the growing body of research on the subject [27,28].

This dependence is conceptualized as a relational pathology expressed through addictive and maladaptive behaviors in dating relationships, characterized by a chronic pattern of seeking to fulfill unmet affective needs. It is specifically described as an excessive need for affection and approval from another person, which is associated with the persistent pursuit of relationships in which individuals experience feelings of incompleteness or emptiness in the absence of the other’s presence or recognition [28].

ED fosters unhealthy bonds characterized by suffering, frustration and fear, leading to abusive and possessive dynamics in relationships [29]. In this context, love can be transformed into an addiction, where the need for another person’s company surpasses the genuine feeling of love. This type of obsessive love entails a constant search for attention and an attempt to control another person’s freedom [30].

ED can lead victims to tolerate abusive behaviors due to a strong emotional attachment to their partner, making it difficult to break the relationship and perpetuating the cycle of abuse. Research [16,31,32] has reported that ED is significantly related to intimate partner violence and that this relationship is more marked in women. That is, women with high ED were more likely to experience mistreatment and abuse in their intimate partner relationships. However, Marcos et al. [33] and Macía et al. [34] found that men reported higher levels of ED and also indicated experiences of victimization, though the type and severity of violence differed between men and women. Despite these results, Urbiola et al. [24] found no significant differences in ED between women and men, mentioning that dependence is an important factor to consider in the context of DaV, regardless of gender and sexual orientation. These findings also suggest that gender may act as a moderator in the relationship between dependence and victimization.

In addition, ED has been consistently associated with poorer mental health indicators during adolescence and young adulthood. Higher levels of ED are linked to greater psychological distress, including emotional discomfort and internalizing symptoms, which may co-occur with maladaptive relational patterns [31,35]. Urbiola et al. [36] and Momeñe et al. [37] mention that ED has serious consequences for mental health; this dependence can trigger internalizing symptoms, especially in women. These sequelae include anxious–depressive symptoms, obsessive thoughts, sleep disorders, and social isolation.

Despite the growing body of literature examining dating violence, emotional dependence and mental health, evidence integrating these variables remains limited, particularly regarding the role of gender. Although previous research has primarily focused on female victims—given their historically higher risk of victimization and poorer mental health outcomes [35,38,39,40,41,42]—recent studies suggest that, in the context of dating violence, men and women may report comparable levels of victimization [16,33,34,43]. However, it remains unclear whether the indirect association between DaV and mental health problems through emotional dependence differs by gender, especially among university students in Latin American contexts. Addressing this gap is particularly relevant in Ecuador, where empirical studies adopting integrative analytical approaches are scarce. Examining these associations jointly may contribute to a more nuanced understanding of relational and psychological risk factors and provide evidence to inform culturally sensitive preventive and educational strategies.

**Figure 1:**
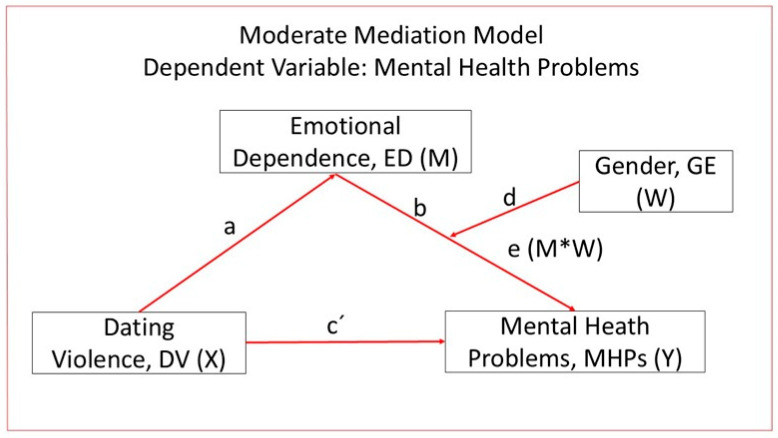
Diagram of the moderated mediation model (PROCESS Model 14, Hayes). Associations between dating violence (DaV; X) and mental health problems (MHPs; Y) through emotional dependence (ED; M), with gender (GE; W) as a moderator. Letters a–e represents the regression paths estimated in the model (a = X→M, b = M→Y, c′ = direct effect X→Y, d = W→Y, e = interaction M × W). The symbol “*” indicates the interaction term between emotional dependence and gender.

Based on previous empirical and theoretical research, a conditional process model was proposed: to evaluate a model examining the association between dating violence (X) and mental health problems (Y) among university students, with emotional dependence (M) as an intervening variable and gender (W) as a moderator (Figure 1). Given the cross-sectional nature of the study, the model does not assume causal effects but rather tests whether the association between dating violence and mental health problems is indirectly associated with emotional dependence, and whether this indirect association varies as a function of gender. This approach allows for the examination of gender-related differences in the strength or direction of the associations among these variables, contributing to a better understanding of how relational dynamics and psychological well-being are interconnected in university students.

Based on this study, three hypotheses were proposed:

**H1:**  *Dating violence (X) is positively associated with mental health problems (Y) in university students.*

**H2:**  *Dating violence (X) is associated with higher emotional dependence (M), which is in turn associated with greater mental health problems (Y).*

**H3:**  *Gender (W) will moderate the relationship between dating violence (X) and emotional dependence (M), or that between emotional dependence (M) and mental health problems (Y), such that the strength or direction of these relationships will vary according to gender*.

## 2. Materials and Methods

### 2.1. Design

This study adopted a quantitative approach, focusing on the numerical examination and statistical analysis of the collected data. It is descriptive and explanatory in nature. Additionally, the research employed a non-experimental, cross-sectional design and was classified as field research, as standardized psychological tests were administered to the study participants.

### 2.2. Participants

The reference population, according to the most recent data from the Ecuadorian higher education system [44], consisted of approximately 60,000 students from the National University of Chimborazo, the Higher Polytechnic School of Chimborazo, and the Central University of Ecuador. A theoretical minimum sample size was estimated using conventional parameters (98% confidence level and ±2% margin of error), yielding a target of 3202 participants. Due to the operational characteristics of the study and voluntary participation, a non-probabilistic convenience sampling strategy was employed. The final sample comprised 3202 Ecuadorian university students who met the inclusion criteria (35.7% men; 64.3% women). Women were overrepresented in the sample, reflecting national trends in Ecuadorian higher education, where approximately six out of ten university students are women, according to official education system reports [45]. Table 1 presents the sociodemographic characteristics of the sample by sex. Most participants were from urban areas, with no significant differences between men and women. Regarding ethnicity, 89.5% of women and 87.1% of men identified as mestizo; however, a higher proportion of men identified as White (2.4%) and Afro-Ecuadorian (1.6%) compared to women (0.7% and 0.4%, respectively). In terms of relationship status, 51.9% of women reported currently having a partner compared to 44.0% of men, whereas 56.0% of men and 48.1% of women reported not currently having a partner but having had one previously. The mean age was similar between groups (women: M = 21.45; men: M = 21.68). Differences were statistically significant for ethnicity and relationship status, while no significant differences were observed for place of origin or age.

### 2.3. Procedure

The purpose of the research was explained to the deans of the faculties at the selected universities, who provided the necessary approvals. Subsequently, the secretaries of each academic program were contacted to obtain the institutional email addresses of legally enrolled students. Eligible students enrolled during the October–March 2023 academic period were invited. Invitations to participate were sent to the students’ institutional email addresses during this same academic period. Student representatives from each course actively assisted in disseminating information about the study, emphasizing its purpose, the voluntary nature of participation, and the exclusive use of data for research purposes. Participants were also informed that the study results would be shared with the student community. No monetary incentives were offered. Data were collected via Microsoft Forms. The platform required all items to be completed before submission, preventing incomplete questionnaires. Additional validation rules were implemented to ensure data quality. As part of this quality control process, 206 questionnaires were excluded due to inconsistent responses or extremely short completion times, indicating unreliable data. All participants provided informed consent prior to participation. The study was conducted in accordance with the Declaration of Helsinki and its subsequent amendments.

### 2.4. Instruments

The Dating Violence Questionnaire (CUVINO) is a validated tool to measure the victimization of Hispanic American youth during their relationship as a couple [46]. This questionnaire has 42 Likert type items, with 5 answer options that range from 0 (“never”) to 4 (“pretty frequently”). Items are arranged among four violence types: psychological, physical, sexual, and instrumental aggression, and each type of violence is arranged among severity levels: mild, moderate, or severe. This instrument comprises questions such as: (a) for psychological violence: “Does your partner humiliate you publicly?”; (b) for physical violence: “Did your partner beat you?”; (c) for sexual violence: “Does your partner insist in touching you in unwanted ways you do not find pleasant?”; (d) for instrumental violence: “Has your partner gotten you in debt?”. The questionnaire’s reliability is high, with Cronbach’s alpha (α) of 0.98 for the global questionnaire. Additionally, the reliability for each type of violence is also high: psychological (α = 0.97), physical (α = 0.85), sexual (α = 0.89), and instrumental aggression (α = 0.88). For analysis, a global score was computed by adding all instrument items, providing a continuous measure of overall victimization in dating relationships.

The Strengths and Difficulties Questionnaire (SDQ-Cas), is a short, 25-item questionnaire that was developed on the London Psychiatry Institute by Robert Goodman [47]. Although originally designed for children and adolescents, the SDQ was used in this study as a dimensional measure of psychological adjustment in young adults, based on the continuity of emotional and behavioral problems across developmental stages and on previous evidence supporting its use in university populations [48]. The university setting offers a relevant context for assessing mental health due to increased vulnerability from high academic demands and greater personal autonomy. The SDQ was administered as a self-report measure for participants aged 18 and older. It consists of 25 items divided into five subscales: (1) Emotional Symptoms, (2) Conduct Problems, (3) Peer Problems, (4) Hyperactivity, and (5) Prosocial Behavior, with five items per sub-scale. For instance, the Emotional Symptoms subscale includes the item “I am often unhappy, down-hearted or tearful”. In contrast, the Conduct Problems subscale includes “I often get angry and lose my temper” and “I am often disobedient at school or university”. Items are rated on a 3-point Likert scale (0 = Not true, 1 = Somewhat true, 2 = Certainly true). For community samples, it is recommended to combine the Conduct Problems and Hyperactivity subscales into a composite Externalizing Problems scale, and the Emotional Symptoms and Peer Problems subscales into an Internalizing Problems scale. The instrument’s total reliability was α = 0.848. The reliability of the emotional problems subscale was α = 0.770, and that of behavioral problems was α = 0.764.

The emotional dependence questionnaire (EDQ) is an instrument created and validated Lemos and Londoño [49] to evaluate the emotional dependence among Hispanic American young people and adults. The instrument is made up of 23 items with 6 answer options: 1 (“completely false on my case”) up to 6 (“it describes me perfectly”). To find if a person is emotionally dependent, the author explains that the cut point employed is the amount between the average and the standard deviation (80.42, that is, 81 total points). The questionnaire encompasses six dimensions: (a) fear of loneliness (e.g., “Do I feel helpless when I am alone?”); (b) separation anxiety (e.g., “Does the idea of my partner leaving me worry me?”); (c) affectionate expression from the partner (e.g., “Do I constantly require expressions of affection from my partner?”); (d) modification of plans (e.g., “If I have made plans and my partner appears, do I alter my plans solely for them?”); (e) attention seeking (e.g., “Do I go out of my way to become the focal point in my partner’s life?”); and (f) borderline expression (e.g., “Am I needy and weak?”). For analytical purposes, the total continuous score of the EDQ was utilized, calculated by summing all item scores. The EDQ has demonstrated high reliability, with a Cronbach’s alpha of 0.927 for the global scale in the original validation study. In the present sample, the instrument also exhibited excellent internal consistency (α = 0.927).

### 2.5. Data Analysis

Initially, reliability analyses were conducted to evaluate the internal consistency of the instruments used in the study prior to subsequent correlational and moderated mediation analyses, followed by a correlational analysis of the study variables. Subsequently, moderated mediation analyses were performed utilizing Hayes’ PROCESS macro for SPSS (2018) [50], which computes indirect effects, standard errors, and 95% confidence intervals (CIs) through bootstrapping. This approach facilitates statistical inference independent of data normality and sample size considerations. Indirect effects were estimated for two moderated mediation models (PROCESS, Model 14) employing 10,000 bootstrap samples, and statistical significance was ascertained by confirming that the 95% CI did not encompass zero.

Assumptions of normality and homoscedasticity were examined using the Kolmogorov–Smirnov and Levene’s tests, respectively, with both tests yielding *p* values greater than 0.05. Nevertheless, as the indirect effect was estimated through bootstrapping, the normality of its sampling distribution was not a prerequisite. Linear relationships between the predictors (DaV, ED, and gender) and the dependent variables (IMHP and EMHP) were assessed via scatterplots, which confirmed linear associations supported by statistical significance (*p* < 0.001). Variance inflation factor (VIF) values below 10 and tolerance values above 0.10 indicated the absence of multicollinearity among the variables.

Four of the five variables in the models (DaV, ED, IMHP, and EMHP) were total scale scores expressed as mean values, whereas gender was coded as 1 = male and 2 = female. No covariates were included in the statistical models, as the analyses were designed to examine the direct relationships among the primary study variables. A post hoc power analysis was conducted using G*Power version 3.1 based on the multiple regression model underlying the moderated mediation analysis. Using the observed coefficient of determination (R^2^ = 0.12), corresponding to an effect size of f^2^ = 0.14, with four predictors and a significance level of α = 0.05, the achieved statistical power with the present sample (N = 3202) was greater than 0.99.

The statistical analyses were carried out with the SPSS version 21.0 for PC statistics package.

## 3. Results

### 3.1. Correlation Analysis of Variables Under Study

Table 2 shows the correlations of all the variables involved in the study. Dating violence (DaV) was directly related to emotional dependence (ED) and to internalizing (IMHP) and externalizing mental health problems (EMHP). Likewise, the ED variable was directly related to IMHP and EMHP. There was a direct correlation between IMHP and EMHP. Finally, the gender variable was directly related to IMHP. All correlations were significant, with *p* < 0.01. 

### 3.2. Moderated Mediation Model—Dependent Variable: IMHP

**Figure 2:**
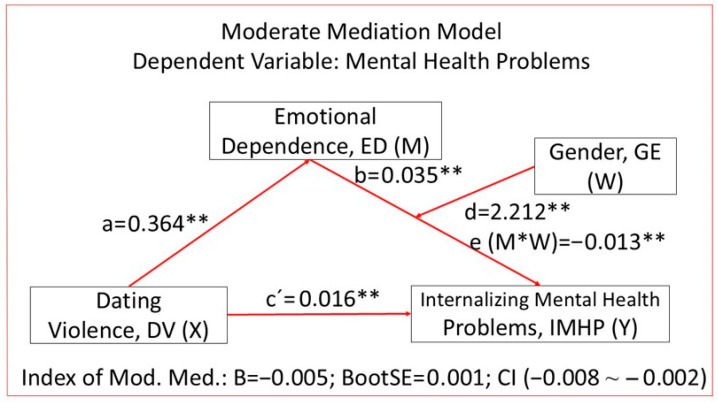
Diagrams and results of the moderated mediation analysis, IMHP. Note: ** *p* < 0.01. The symbol “*” indicates the interaction term between emotional dependence (M) and gender (W).

Figure 2 shows the data for the moderated mediation model. The model meets the assumptions for the application of mediation analysis: significant relationships between the independent variable and the dependent variable, between the independent variable and the mediator, and between the mediator and the dependent variable.

Table 3 shows the data from the moderated mediation analysis. Regarding the results on mediation, we can state that the regression analysis between the mediating variable ED and the independent variable DaV showed a significant positive relationship (a: B = 0.364; β = 0.493; SE = 0.011; *p* < 0.001), explaining the model with 24% of the variance of the mediating variable ED. The results of the multiple linear regression analysis considering DaV and ED as predictor variables showed a significant positive relationship between DaV and the dependent variable IMHP (c’: B = 0.016; β = 0.273; SE = 0.001; *p* < 0.001) and between ED and IMHP (b: B = 0.035; β = 0.237; SE = 0.006; *p* < 0.001). The statistical significance of the indirect effects was demonstrated by checking that the established confidence interval (95% CI) did not contain the value 0, finding a statistically significant indirect effect (B = 0.005; BootSE = 0.001; Boot = 95%, CI [0.003~0.007]).

**Figure 3:**
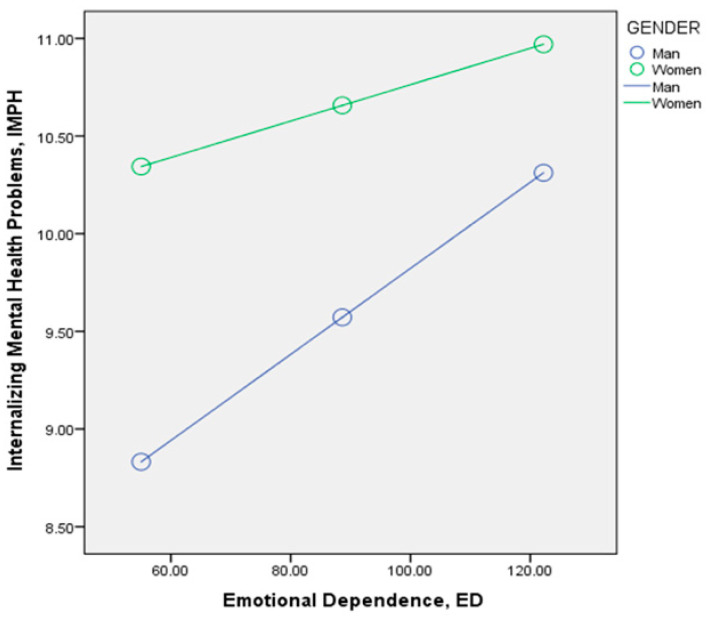
Moderating effect of gender on the relationship between emotional dependence (ED) and internalizing mental health problems (IMHP).

Regarding the results of moderation, the linear regression of the moderating variable GE on the dependent variable IMPH showed a significant positive relationship (d: B = 2.212; β = 0.151; SE = 0.347; *p* < 0.001). Most importantly, the interaction of the mediating variable ED and the moderating variable GE obtained significant values (e: B = −0.013; SE = 0.004; *p* = 0.004), so we can consider that gender interferes with the effect of emotional dependence (ED) on internalizing mental health problems (IMPH). As we can see in Figure 3, the gender variable had a greater moderating effect for males (B = 0.022; SE = −0.013; *p* < 0.001) than for females (B = 0.009; SE = 0.002; *p* < 0.3001), although both were significant. The impact of emotional dependence on internalizing problems was stronger in men than in women.

Regarding moderated mediation, or how the moderating variable GE influences the indirect effect, conditional results on the indirect effects indicate that the indirect effect was larger for men (B = 0.008; BootSE = 0.001; Boot 95% CI [0.005~0.011]) than for women (B = 0.003; BootSE = 0.001; Boot 95% CI [0.002~0.005]). Finally, the moderate mediation index was significant (B = −0.005; BootSE = 0.001; Boot 95% CI [−0.008~−0.002]), so we can conclude that there is moderate mediation and that the indirect effect of dating violence on internalizing mental health problems through emotional dependence is moderated by gender.

### 3.3. Moderated Mediation Model—Dependent Variable: Externalizing Mental Health Problems (EMHP)

Table 4 shows the data from the moderated mediation analysis. Regarding the results on mediation, we can point out that the assumptions for a mediation analysis were not met, since there was no significant relationship between the mediating variable ED and the dependent variable EMHP (b: B = 0.007; β = 0.139; SE = 0.007; *p* = 0.328). Regarding the results on moderation, we can point out that the assumptions for a moderation analysis were not met, since there was no significant relationship between the moderating variable GE and the dependent variable EMHP (d: B = 0.010; β = 0.003; SE = 0.129; *p* = 0.936); neither the interaction of the mediator variable ED and the moderator GE obtained significant values (e: B = 0.001; SE = 0.004; *p* = 0.864). In short, the hypothesis that there is moderate mediation and that the indirect effect of dating violence on externalizing mental health problems through emotional dependence is moderated by gender was not met.

## 4. Discussion

Research shows that DaV and ED are closely linked, aligning with earlier studies [16,31,33] suggesting that this violence may be part of a cycle of abuse in which ED is present. Additionally, both DaV and ED are interconnected and may be associated with poorer MH. The results confirmed hypotheses one and two, but hypothesis three was only partly supported, as gender influenced internalized mental health issues but did not significantly affect externalized problems. The following sections analyze these findings in more detail to better understand the observed differences.

### 4.1. DaV, ED, and IMPH

The results indicate that both DaV and ED were significantly associated with IMPH. DaV was associated with internalizing disorders, and this association appeared stronger in participants with higher ED, with ED functioning as a mediating variable, with higher levels of ED being associated with stronger associations between DaV and IMPH.

Several studies examined the relationship between DaV and MH [1,51,52,53,54], as well as between ED and MH or between ED and MH [31,36], but few investigations have simultaneously addressed both factors [36,55]. Therefore, research studying these three variables together is limited. Consequently, research exploring these three variables collectively remains scarce. This study concurs with the findings of Momeñe et al. [29], who indicated that ED and violence were significantly associated with heightened psychopathological symptoms, including depression, anxiety, interpersonal sensitivity, obsessive-compulsive behaviors, and paranoid ideation. Similarly, Ponce-Díaz [56] found that individuals with ED who experienced intimate partner violence exhibited elevated levels of distressing emotions, such as sadness, fear, guilt, frustration, and uncertainty, which was associated with lower perceived happiness, life satisfaction, and overall mental health.

Nevertheless, this research underscores the role of ED; the experience of violence in dating relationships has been associated with higher levels of IMPH. Furthermore, individuals exhibiting high levels of ED demonstrate a more pronounced correlation between ED and adverse outcomes when subjected to violence. ED may be associated with greater susceptibility to experiences of violence, as their emotional well-being is intricately connected to the partner relationship [16], which is linked to greater vulnerability to internalizing disorders.

In other words, internalizing problems may be associated with constant exposure to stressful and abusive situations, which is related to the emotional and psychological stability of the victim. The person who experiences violence in their dating relationship and exhibits ED toward their abusive partner may report a greater tolerance of abusive behavior and continued involvement in the relationship. This dependence is associated with a stronger relationship and is related to anxiety and depressive symptoms [55].

### 4.2. Gender, ED, and IMHP

The data from this study also revealed that gender not only has an association with IMHP, but also moderates the relationship between ED and these problems. In particular, this dependence shows a stronger association with IMHP in men. This information contradicts existing literature [1,31,51], which suggests that ED has a more pronounced effect on MH in women compared to that in men.

One possible explanation for this discrepancy is that women, irrespective of their ED status, are more vulnerable to mental health issues due to sociocultural and biological influences [40,41,57]. Women generally tend to experience and report elevated levels of anxiety and depression relative to men, which may diminish the apparent differences attributable to ED when compared to women without ED.

On the other hand, men with higher levels of ED may show stronger associations with IMHP, which may be related to more limited emotional expression and a lower likelihood of seeking help. This reduced emotional expression has been associated with greater difficulty managing negative emotions, increasing the risk of developing mental health problems such as depression [58] and anxiety.

Gender norms that value strength and self-reliance in men may contribute to a greater accumulation of stress and untreated health problems [59], which may be linked to higher levels of IMHP in men with ED. In addition, ED in men may be related to a greater perception of loss of control and autonomy, which may intensify internalized feelings.

The moderated mediation model in this study indicates that ED mediates the relationship between DaV and IMHP, while gender moderates the second half of the mediated pathway: “DaV–ED–IMHP”. This model suggests that, in the context of DaV, IMHP tend to be higher among men with high ED.

### 4.3. DaV, ED, and EMHP

Results also reveal that it cannot be claimed that the indirect effect of DaV on EMHP through ED is moderated by gender. This implies that the moderate mediation hypothesis was not met in this case. However, a few studies [34,60] claim that ED is also correlated with behavioral problems, aggressiveness, and impulsivity in men. For example, Marcos et al [33] found that men with high ED tended to present aggressive behaviors and disruptive behaviors in their partner relationships. That is, ED in men was significantly correlated with greater belief in romantic love myths and sexism, which contributed to the perpetration of DaV.

However, there is no strong evidence to show that men with ED are more likely to experience greater EMHP. Therefore, men and women who experience DaV and have high ED may present with EMHP in a similar manner. In other words, ED acts as a universal mediator in the relationship between DaV and EMHP, without gender significantly altering this dynamic.

Highly ED young people (both male and female) may react aggressively when they feel that their relationship is threatened. This aggressiveness can manifest itself in disruptive behaviors in their social environment, such as fights, heated arguments, and acts of physical or verbal violence [61]. ED is characterized by an excessive need for affection and approval from a partner, which can lead to a distorted perception of threats to the relationship [28]. When these young people perceive that their relationship is in jeopardy, they may experience high levels of anxiety and fear of abandonment. These feelings can trigger aggressive responses as a defense mechanism to try to control the situation and avoid losing a partner. Aggressiveness may be a way of expressing frustration and despair at the possibility of being left alone [62].

In addition, men and women with high ED may exhibit impulsive and disruptive behaviors as a way of dealing with emotional stress. Impulsivity can lead them to make rash decisions and act without thinking through the consequences of their actions [34]. This may include behaviors such as stalking, constant surveillance of the partner as a way of emotionally manipulating the partner so that he or she does not leave them.

### 4.4. Constraints

This study is not without limitations. First, women were overrepresented in the study; therefore, any generalizations involving gender should be interpreted with caution. This overrepresentation may be partly explained by recent changes in educational demographics [45]. Second, the cross-sectional quantitative design did not allow for an examination of temporal ordering or causal relationships, nor did it permit an in-depth exploration of the participants’ individual experiences or contextual factors. Third, social desirability bias was not controlled, which may have led the participants to respond in ways perceived as more socially acceptable rather than fully accurate or honest. This limitation is particularly relevant in research on relationship violence, as individuals may feel uncomfortable or embarrassed when reporting violent or abusive behaviors. Fourth, omitted variable bias cannot be ruled out due to limited covariate adjustment, and generalizability may be further constrained by the characteristics of the sample. Fifth, the use of a non-probabilistic convenience sample may limit the generalizability of the findings to the broader university population. These limitations should be considered when interpreting the findings. Future research would benefit from longitudinal or multi-wave designs to clarify temporal ordering, strengthen causal inference, and provide a more nuanced understanding of the associations among dating violence, emotional dependence, and mental health outcomes.

## 5. Conclusions

This research shows that ED is associated with greater vulnerability to intimate partner violence, which is associated with higher levels of mental health problems. Relationship violence may be part of a cycle of dependency, making it difficult for the victim to leave the abusive relationship. Understanding these dynamics may inform the development of interventions that address both violence and dependence, improving the mental health of affected individuals and reducing the incidence of internalized problems.

Male victims of violence with ED presented higher levels of internalized problems, which underscores the importance of further studying gender in this context. In countries such as Ecuador, interventions tend to be individualistic and focused exclusively on women [63], which is a mistake. Interventions should be integrative and consider both genders, as men can also manifest high levels of dependency and suffer from its consequences. Ignoring this reality may limit the effectiveness of prevention and treatment strategies, contributing to the persistence of unaddressed mental health problems in the male population.

Finally, gender was not found to be a significant moderator of violence and ED in relation to externalizing problems. The lack of moderation by gender suggests that both men and women may experience externalizing problems stemming from violence and ED, and that other factors, such as cultural, socioeconomic or individual context, may play a more important role in these dynamics. Therefore, future research should explore these additional variables to develop more precise and effective interventions.

## Figures and Tables

**Table 1:** Demographic characteristics of the groups of women and men.

	Women (n: 2060)		Men (n: 1142)		χ^2^-Value
	n	%	n	%	
	Place of origin				2.69
Urban Area	1422	69.0	820	71.8	
Rural Area	638	31.0	322	28.2	
	Ethnicity				28.40 **
Mestizo	1844	89.5	995	87.1	
Indigenous	177	8.6	92	8.1	
Afro-Ecuadorian	9	0.4	18	1.6	
Montubio	16	0.8	10	0.9	
Caucasian	14	0.7	27	2.4	
	Couple				18.13 **
Currently in a relationship	1069	51.9	502	44.0	
Not currently in a relationship, but has been in one before	991	48.1	638	56.0	
Age	M	DS	M	DS	t value
	21.45	2.88	21.68	2.71	1.43

Note: ** *p* < 0.01.

**Table 2:** Results of the correlation analysis of the variables under study.

	1. GE	2. DaV	3. ED	4. IMHP	5. EMHP
1. GE	-	0.004	0.021	0.151 **	0.003
2. DaV		-	0.493 **	0.273 **	0.171 **
3. ED			-	0.237 **	0.139 **
4. IMHP				-	0.630 **
5. EMHP					-
M	-	56.40	88.62	10.27	7.68
SD	-	45.55	33.59	3.53	3.56
%	64.3%	-	-	-	-

GE = gender (1 = male, 2 = female); DaV = dating violence; ED = emotional dependency; IMHP = internalizing mental health problems; EMHP = externalizing mental health problems; ** *p* < 0.01.

**Table 3:** Results of the moderate mediation analysis, IMHP (PROCESS, model 14).

**Direct Relations**	**Path**	**Β**	**SE**	***p***
Effect DaV–ED	A	0.364	0.011	0.000
Effect ED–IMHP	B	0.035	0.006	0.000
Direct effect DaV–IMHP	c’	0.016	0.001	0.000
Effect GE-IMHP	D	2.212	0.347	0.000
Interaction Effect ED × GE–IMHP	E	−0.013	0.004	0.001
ED effect model (F = 1029.036; *p* < 0.001; R^2^ = 0.24)				
IMPH effect model (F = 102.728; *p* < 0.001; R^2^ = 0.12)				
**Indirect Relationships**	**Β**	**BootSE**	**IL**	**LL**
Mediation Indirect Relationships				
	0.005	0.001	0.003	0.007
Moderated Indirect Relationships				
Indirect effect, male	0.008	0.001	0.005	0.011
Indirect effect, female	0.003	0.001	0.0002	0.005
Index of Moderated Mediation	−0.005	0.001	−0.008	−0.002

Note. DaV = dating violence; ED = emotional dependence; IMHP = internalizing mental health problems; GE = gender (1 = male, 2 = female). PROCESS Model 14; unstandardized coefficients (Β) are reported. BootSE = bootstrap standard error; IL = lower limit; LL = upper limit.

**Table 4:** Results of the moderate mediation analysis, EMHP (PROCESS, model 14).

Direct Relationships	Path	β	SE	*p*
Effect DaV–ED	A	0.364	0.011	0.000
Effect ED–EMHP	B	0.007	0.007	0.328
Direct effect DaV–EMHP	c’	0.011	0.002	0.000
Effect GE-EMHP	D	0.010	0.129	0.936
Effect of Interaction ED × GE–EMHP	E	0.001	0.004	0.864
ED effect model (F = 1029.036; *p* < 0.001; R^2^ = 0.24)				
EMHP effect model (F = 27.303; *p* < 0.001; R^2^ = 0.03)				

Note. DaV = dating violence; ED = emotional dependence; EMHP = externalizing mental health problems; GE = gender (1 = male, 2 = female). PROCESS Model 14; unstandardized coefficients (β) are reported.

## Data Availability

The datasets generated during and/or analyzed during the current study are available from the corresponding author on reasonable request.

## Abbreviations

The following abbreviations are used in this manuscript:

DaV	Dating violence
ED	Emotional Dependence
MH	Mental health
IMHP	Internalizing mental health problems
EMHP	Externalizing mental health problems
